# Task-dependent calibration of auditory spatial perception through environmental visual observation

**DOI:** 10.3389/fnsys.2015.00084

**Published:** 2015-06-02

**Authors:** Alessia Tonelli, Luca Brayda, Monica Gori

**Affiliations:** Robotics, Brain and Cognitive Sciences Department, Fondazione Istituto Italiano di TecnologiaGenoa, Italy

**Keywords:** audio, vision, bisection, multisensory, calibration, space perception, echoes

## Abstract

Visual information is paramount to space perception. Vision influences auditory space estimation. Many studies show that simultaneous visual and auditory cues improve precision of the final multisensory estimate. However, the amount or the temporal extent of visual information, that is sufficient to influence auditory perception, is still unknown. It is therefore interesting to know if vision can improve auditory precision through a short-term environmental observation preceding the audio task and whether this influence is task-specific or environment-specific or both. To test these issues we investigate possible improvements of acoustic precision with sighted blindfolded participants in two audio tasks [minimum audible angle (MAA) and space bisection] and two acoustically different environments (normal room and anechoic room). With respect to a baseline of auditory precision, we found an improvement of precision in the space bisection task but not in the MAA after the observation of a normal room. No improvement was found when performing the same task in an anechoic chamber. In addition, no difference was found between a condition of short environment observation and a condition of full vision during the whole experimental session. Our results suggest that even short-term environmental observation can calibrate auditory spatial performance. They also suggest that echoes can be the cue that underpins visual calibration. Echoes may mediate the transfer of information from the visual to the auditory system.

## Introduction

The visual system is the most accurate sense to estimate spatial properties. Many studies involving adult individuals support this idea, showing that when the spatial locations of audio and visual stimuli are in conflict, vision usually dominates, generating the so-called “ventriloquist effect” (Warren et al., 1981; Mateeff et al., 1985). This effect is possibly due to an optimal combination of cues performed by the human brain, where modalities are weighted by their statistical reliability. Vision dominates over audition in localization tasks (Alais and Burr, 2004). When visual and auditory systems are simultaneously presented to get spatial information, the final multisensory estimate tends to be more precise than either unisensory estimate (Clarke and Yuille, 1990; Ghahramani et al., 1997; Ernst and Banks, 2002; Alais and Burr, 2004; Landy et al., 2011). Interestingly, vision can interact with audition even when a visual stimulus is not provided during an auditory task: specifically. For example, although the angle of incidence of a sound source can be estimated with the use of only auditory cues, performance improves when vision is also present (Jackson, 1953; Shelton and Searle, 1980). A recent study by Tabry et al. (2013) has shown that the mere possibility of observing the setup by keeping eyes open during auditory horizontal and vertical localization tasks can improve audio accuracy, even if no visual cues of the stimuli are provided.

Another example of the connection between audio-spatial and visuo-spatial information is given by a technique used by some blind people, called echolocation. Some studies have shown that this technique can operate as a crude substitute for vision, because some purely visual phenomenona, such as size constancy (Milne et al., 2015) or size-weight illusion (Buckingham et al., 2015), are present in expert echolocators, who use echoes to navigate in unknown environments.

However, it is still unknown which are the visual cues that allow an improvement in audio-spatial tasks, nor it is understood how long visual cues should last to determine such improvement. As well, it is unknown whether this phenomenon can be task-specific, i.e., if audio-spatial abilities are improved in general or if the influence of vision depends on the complexity of the audio task. Can it be argued that increased audio-spatial abilities are due to a transfer of information from the visual to the auditory system? Which is the information that is transferred? Is vision more informative for some aspects than for others?

In this paper we tested two audio tasks under various environmental conditions and visual feedbacks to answer these questions. In particular we investigated: (i) whether the environmental visual cues (i.e., prior short observation of the environment and full vision during the tasks) can improve auditory precision; (ii) whether this improvement is task-specific; and (iii) which are the environmental cues that mediate the auditory improvement due to the interaction between vision and audition.

To investigate the first point about whether environmental visual cues can improve auditory precision we tested a sample of blindfolded sighted participants twice: the first time with no visual input of the environment where the auditory task was performed; the second time after they observed for 1 min the environment. We compared the performance with no visual input of the environment with that with 1 min observation. We also tested a different group of sighted participants, who performed the two tasks with a full vision of the room but without being blindfolded. We compared the performance of this last sample with the other. Our hypothesis was that if the visual cues coming from the environmental observation can help to improve the auditory precision, then the improvement should occur at least with full vision and possibly with short-term observation.

The second question was about whether auditory precision improvement was task-specific. We tested all participants in two audio tasks: the minimum audible angle (MAA) task and the spatial bisection. In the MAA task the participant had to judge which of the two sounds generated by an array of loudspeakers was more from the right. Instead, in the spatial bisection task the participant heard three sounds coming from three distinct locations and had to judge whether the second sound was closer to the first or third sound coming from the array. The difference between these two tasks is that the spatial bisection task requires subjects to encode the position of three sounds, remember them over a period of 1 s and compare their remembered positions. Contrarily, in the MAA task, the subject has to compare the position of the two sounds relatively to the subject’s position. Moreover while MMA requires estimating a relation of order between two acoustic directions; bisection requires estimating a relation of order between two estimated acoustic distances. To summarize, while the space bisection requires a Euclidian representation of space and involves higher abstraction capabilities, for the MMA task a topological representation of space is sufficient.

Moreover, we chose these two tasks because we recently reported that the visual information is fundamental for the bisection task and not essential for the MAA task (Gori et al., 2014). A visual dominance over audition during development was observed for the space bisection task (Gori et al., 2012), while the absence of visual input in congenitally blind individuals negatively impacts their performance on audio space bisection tasks (Gori et al., 2014). However the absence of vision does not affect the ability of performing the MAA task in visually impaired individuals (Gori et al., 2014; in agreement with Lessard et al., 1998).

The apparent influence of the visuo-spatial knowledge on space bisection tasks leads to our second hypothesis: if the environmental visual information can improve acoustic spatial precision, then the improvement should be bigger for the space bisection task than for the MAA task.

With regard to the third point, which consists in investigating the environmental cues that possibly mediate the auditory improvement after observation, we replicated all audio tests in an anechoic room. In such a room, the walls absorb part of the sound energy; therefore the auditory system almost exclusively acquires the direct path of the sound, i.e., not reflected by walls. Conversely, in the normal room, a wall reflects sounds and generates echoes. Our hypothesis is that if the interpretation of echoes is triggered by visual observation, an improvement of acoustic precision should occur only in the normal room, while not in the anechoic chamber.

## Materials and Methods

### Participants

We measured auditory spatial discrimination in 33 sighted individuals with normal or corrected to normal vision (an average age of 28, 5 years, with 18 females and 15 males), all with normal hearing (assessed by Ear Test 1.0 software) and no cognitive impairment. All participants gave informed consent before starting the tests. The study was approved by the ethics committee of the local health service (*Comitato Etico, ASL3, Genova*).

### Apparatus and Stimuli

**Figure 1:**
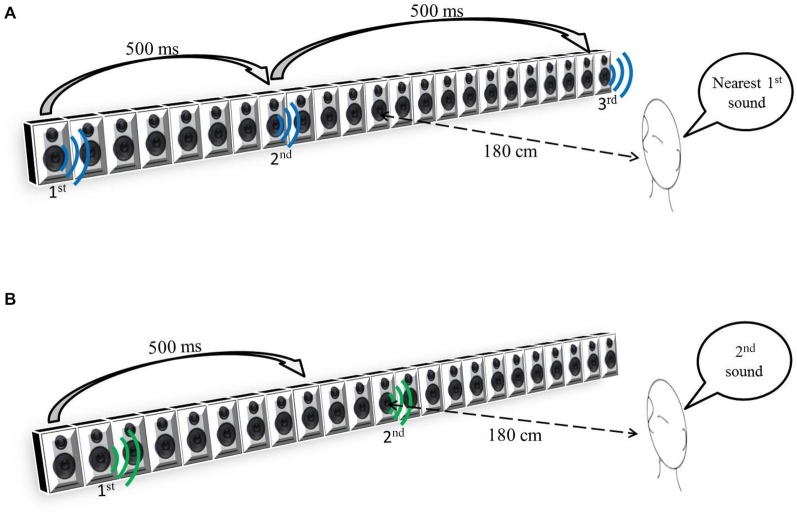
**(A)** Space Bisection Task. **(B)** Minimum audible angle (MAA) task.

The participants sat 180 cm away from the center of a bank of 23 speakers, 161 cm long (see Figure 1), and spanning ±25° of visual angle.

During the auditory space bisection task, three stimuli, each having a duration of 75 ms, were presented at interval of 500 ms (see Figure 1A). The first stimulus was always at −25°, the third always at +25° and the second at an intermediate speaker position which was determined by QUEST (Watson and Pelli, 1983), an adaptive algorithm which estimates the best stimulus value to be presented after each trial, given the current participant’s estimate. To ensure that a wide range of positions was sampled, that estimate was jittered by a random amount, drawn from a Gaussian distribution of space covering the full width of the loudspeaker’s array, and the nearest speaker to that estimate chosen. In the MAA task, two 75 ms pink noise (Will and Berg, 2007) stimuli were presented with a 500 ms interval. One sound came from the central loudspeaker (12th speaker) and the other one at a random distance from center on its left or on its right (Figure 1B). Also in this case, the QUEST algorithm determined the position of the second stimulus. For both tasks, the proportion of rightward responses was calculated for each speaker distance. Gaussian functions by means of the Maximum Likelihood method were used to estimate both the mean, or PSE (Point of Subjective Equality), and the standard deviation, or JND (Just Noticeable Difference). The standard deviation of the fit was taken as an estimate of the threshold, indicating the precision of the task.

To better generalize our results, in 15 participants we used three different sound sources (randomized across trials), all with a 75 ms duration and a 60 dB SPL intensity (measured at the participant’s position): a 500 Hz sound (for which interaural time differences are more important for sound localization); a 3000 Hz sound (for which interaural level differences are more important); and pink noise (ranging from 0 to 5 KHz) for which both are important. As the precision in sound localization varied very little among the three sounds, only pink noise burst data were considered.

### Procedure

Two audio spatial tasks were considered: an auditory space bisection task and a MAA task. The entire group of participants were divided into three groups. The first group (composed of 11 participants) performed four audio tasks (two times the bisection task and two times the MMA task) in an anechoic chamber (3 m × 5 m), the second groups (composed of 11 participants) performed four audio tasks (two times the bisection task and two times the MAA task) in a normal room (7, 20 m × 3, 5 m). The participants of these two groups were blindfolded before entering the room; during the first two audio tasks (one audio bisection and one MAA task), they had no notion of the room or the acoustic stimulation setup. After having performed both the audio tasks, the participants were allowed to remove the blindfold and observe for 1 min the room: in one case an anechoic chamber (first group) and in the other case a normal room (second group). Afterwards they were blindfolded again to repeat both audio tasks again. The last group (composed of 11 participants) was not blindfolded, so they had a full vision of the room and the setup during the tasks. They performed two audio tasks (one time the bisection task and one time the MAA task) just on the normal room. For all the groups the bisection and MAA tasks were presented in a random order.

In the auditory space bisection, participants reported verbally whether the second sound was spatially closer to the first sound (produced by the first speaker on the left, number 1) than the last sound (produced by the last speaker on the right, number 23). Each subject performed 60 trials.

In the MAA task, the participants had to verbally report which sound was more from the right, choosing between the first or the second sound. Each subject performed 60 trials for each task.

## Results

**Figure 2:**
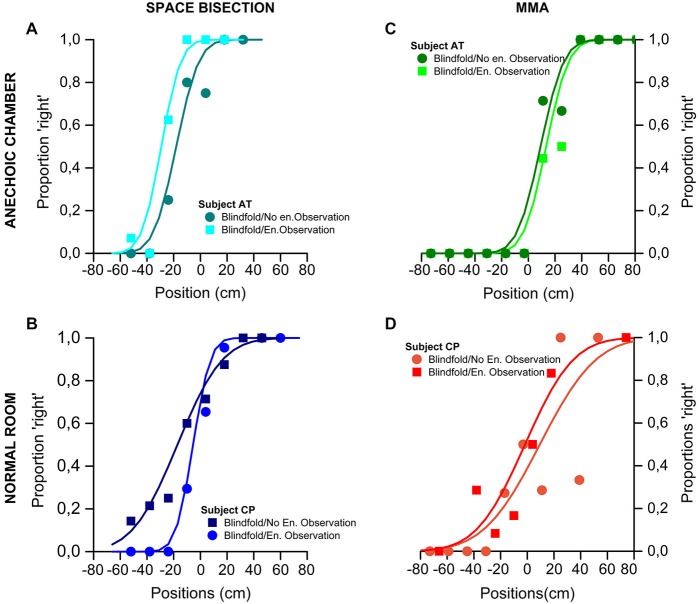
**Results of the Space Bisection Task and MAA of two participants, one for each group (normal room and anechoic chamber) as example. (A,B)** Space bisection: proportion of trials judged “closer to the right sound source”, plotted against speaker position (in cm). The area of the dots is the proportion of trials at that position, normalized by the total number of trials performed by each participants. At the top-left the results obtained in the anechoic chamber by participant AT **(A)**; at the bottom-left the results obtained in the normal room by participant CP **(B)**. Both sets of data are it with the Gaussian error function. **(C,D)** MAA: proportion of trials where the second of a two-sound sequence was reported to the right of the first, plotted against difference in speaker position. At the top-right the results obtained in the anechoic chamber by participant AT **(C)**; at the bottom-right the results obtained in the normal room by participant CP **(D)** Again the fits are the Gaussian error function.

Figure 2 show psychometric functions of the proportion of trials judged “closer to the right sound source”, plotted against speaker position (in cm). On the top of the figure are shown the results obtained by one of the participant took as an example of the global trend in the anechoic chamber for the space bisection (Figure 2A) and the MAA (Figure 2C). In the same way, on the bottom, the Figure 2 show the results of one of the participant for the normal room in the space bisection (Figure 2B) and the MAA (Figure 2D).

**Figure 3:**
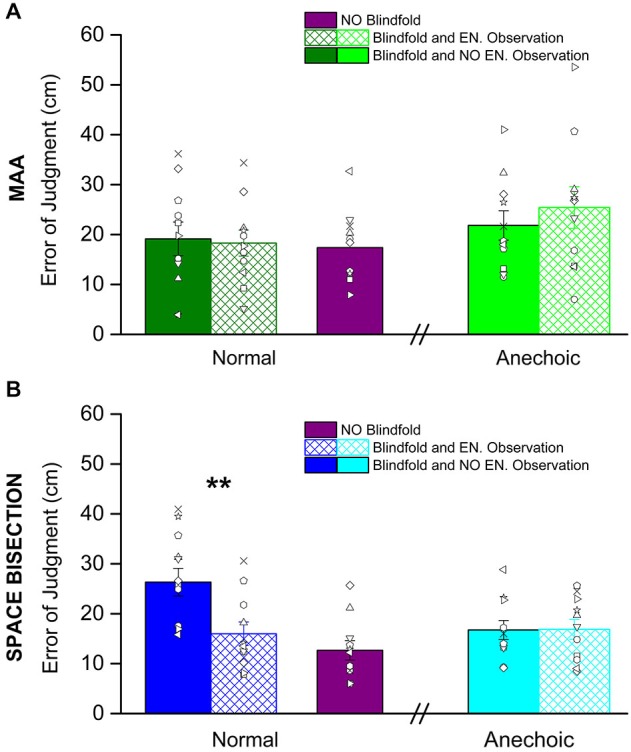
**Shown here are the average precision thresholds obtain in the MAA (A) and Space Bisection (B) tasks. (A)** The dark green bars, on the left, represent the average precision thresholds obtained in the normal room before (fill in dark green bar) and after (reticulus dark green bar) environmental observation. On the right the light green bars are the average precision thresholds obtained in the anechoic chamber before (fill in light green bar) and after (reticulus light green bar) environmental observation. The violet bar is the average precision obtained by the subject in full vision in the normal room. The dots represent individual data. **(B)** For the space bisection, dark blue bars, on the left, represent the average precision thresholds obtained in the normal room before (fill in dark blue bar) and after (reticulus dark blue bar) environmental observation. On the right the light blue bars are the average precision thresholds obtained in the anechoic chamber before (fill in light blue bar) and after (reticulus light blue bar) environmental observation. Also in this case the violet bar represent the average precision obtained by the subject in full vision in the normal room. The dots represent individual data. (**) Indicates a significant difference of precision between before and after environmental observation in the normal room (*p* < 0.01).

Figure 3 shows the thresholds obtained before and after having observed the environment, it also shows the performance with eyes open for both the tasks: the MAA (Figure 3A) and the space bisection (Figure 3B). In both figures, the solid colors refer to the performance before room observation, while the colors with reticulus refer to the performance after the room observation.

We conducted a mixed model 2-way (2 × 2) ANOVA for both MAA and Space Bisection tasks with a between factor, *room kind* (normal room vs. anechoic chamber), and within factor, *room observation* (before environmental observation vs. after environmental observation). For the space bisection task the ANOVA revealed significant main effect for both factors, *room observation* (*F*_(2,22)_ = 6.55, *p* < 0.02) and *room kind* (*F*_(2,22)_ = 7.35, *p* < 0.01). It has been observed a significant *room observation* × *room kind* interaction (*F*_(4,11)_ = 6.86, *p* < 0.01). Then we ran Student’s *t*-test that indicate a significant difference between the groups who performed the space bisection task in the normal room and anechoic chamber before observing the room (two tailed two-sample *t*-test, *t*_(20)_ = 3.44, *p* < 0.01) and in the normal room between before environmental observation and after environmental observation (two tailed pair-sample *t*-test, *t*_(10)_ = 5.46, *p* < 0.001). On the other hand, for the MAA, no significant effect was found (*room observation*, *F*_(2,22)_ = 0.48, *p* = 0.49; *room kind*, *F*_(2,22)_ = 1.49, *p* = 0.28; *room observation* × *room kind F*_(4,11)_ = 0.506, *p* = 0.481).

No significant difference was obtained in the precision after environmental observation and full vision (violet bars) for the space bisection task (two tailed two-sample *t*-test, *t*_(20)_ = 1.279, *p* = 0.27) and for the MAA (two tailed two-sample *t*-test, *t*_(20)_ = 0.257, *p* = 0.799).

No change was observed in the localization bias (PSE) for both groups and tasks (bisection task: 2-ways (2 × 2) ANOVA with factors *room observation*—*F*_(2,22)_ = 0.79, *p* = 0.38—and *room kind*—*F*_(2,22)_ = 1.48, *p* = 0.23—and *room observation* × *room kind* interaction, *F*_(4,11)_ = 0.088, *p* = 0.77; MAA task: 2-ways (2 × 2) ANOVA with factors *room observation*—*F*_(2,22)_ = 0.373, *p* = 0.545—and *room kind*—*F*_(2,22)_ = 1.91, *p* = 0.175—, and *room observation* × *room kind* interaction, *F*_(4,11)_ = 0.001, *p* = 0.97).

## Discussion

Recent works suggest that vision can interact with the auditory modality even when visual information is not useful for the auditory task, by improving the accuracy of auditory localization judgments (Jackson, 1953; Shelton and Searle, 1980; Tabry et al., 2013). For example acoustic performance has been found to be better when participants were allowed to observe the setup by keeping eyes open even if no visual cues were provided (Tabry et al., 2013). Thus even the simple observation of the setup and the environment during the task can improve auditory performance.

Why does this process occur? Which are the visual cues used by the visual system that allow for such an auditory improvement?

In this paper we investigated these issues by studying: (i) the environmental visual cues that are involved in auditory precision improvement; and (ii) whether this improvement is task related.

We tested the first point by asking the participants to perform two audio tasks twice. The first time the tasks were performed without observing the room; the second time, after having observed the room for 1 min. The results suggest that the observation of the environment for a brief period improves the auditory space precision and that the improvement is environment dependent. The improvement was only found after the observation of a natural environment, while, when the test was replicated in an anechoic chamber, no improvement was obtained. Besides, the improvement was task dependent. Two tasks were tested: a MAA task and an audio spatial bisection task; the improvement was observed only for the space bisection task but not for the MAA.

The first question that arises from these results is why the improvement is task-specific, giving that it occurs only for the audio space bisection task. We think that this specificity can be related to the role of visual information on the calibration of the auditory system.

Most recent works on multi-sensory interactions concentrated on sensory *fusion*, investigating the efficiency of the integration of information from different senses. Equally important, but somewhat neglected aspect, is sensory *calibration*.

Our idea is that, while *precision* has the highest weight for sensory integration, the most important property for sensory calibration is *accuracy*. Precision is a relative measure defined as the degree of reproducibility or repeatability between measurements, usually defined as the standard deviation of the distribution. Accuracy, conversely, is defined in absolute terms as the vicinity of a measurement to its true physical value.

We have recently observed that during an audio-visual bisection task, sighted children show a strong visual dominance before multisensory integration occurs (Gori et al., 2012). It is reasonable that for the audio bisection task, the sense of vision is the most accurate for estimating the space. Therefore, it may be used to calibrate the audio system for this spatial task. An important question inferring from our cross-modal calibration theory is: what happens when the calibrating sense is impaired or absent, as is the case of visually impaired adults? We recently tackled this question by testing blind adults in an spatial audio bisection task demonstrating that, in absence of visual input, they have deficits in understanding the spatial relationship between sounds (Gori et al., 2014). The audio deficit was not observed, in agreement with previous studies (Lessard et al., 1998), for the MAA task.

Several physiological works confirm that vision is fundamental for some kind of auditory spatial localization: a series of experiments on animals have documented that displacing vision (Knudsen and Knudsen, 1985) or producing total visual deprivation (King and Carlile, 1993) often lead to systematic and persistent biases in auditory tasks. In the same way, transitory visual distortions in humans produce dramatic changes in auditory spatial maps (Recanzone, 1998; Zwiers et al., 2003).

On the basis of this evidence we can infer that the visual environmental information is not directly involved in the calibration of acoustic system in tasks such as the MAA. This idea would explain why we found a specific audio improvement after environment observation only for the audio bisection task and not for the MAA task.

A second interesting result is that: (i) before the environment observation, audio space bisection performance was worse in the normal room than in the anechoic room; and (ii) after the environment observation, an audio improvement was observed in the normal room and not in the anechoic room. Why performance for the space bisection task did not improve in the anechoic chamber and why it was worse before environment observation in the normal room than in the anechoic chamber? The observed null effect of the short environmental observation in the anechoic room might have been caused by a ceiling effect, i.e., performance was best already before room observation. However, this was not the case in the normal room, suggesting an alternative interpretation: in an anechoic chamber part of the energy of sounds produced by the loudspeakers is absorbed by the walls, therefore the hearing system acquires almost exclusively the direct sound. This is not true in the normal room, where the sound produced by the speakers is reflected by the walls, therefore producing echoes. This results in stimuli with scattering patterns or spectral coloration, or both, which are as much different as source locations are far apart. These echoes add perceptual information to the direct path of the sound, which may not be immediately interpretable without visual input, therefore causing a mismatch and worse performance in the normal room than in anechoic condition. However, the visual system could help the auditory system to compensate for such mismatch and obtain performance again comparable to those obtained in anechoic condition.

For similar reasons, observing an anechoic room does not improve acoustic precision because visual knowledge of the room structure is by no means related to any acoustic cue. Obtaining improvements in both rooms (or in the anechoic room only) would have supported the hypothesis that vision helps in estimating mainly the direction of arrival of acoustic direct paths, i.e., the only cue present in an anechoic room. However, this did not happen, supporting instead the hypothesis that visual cues related, even if implicitly, more to a global footprint given by room acoustics than to the local and specific acoustical feedback of our stimulation setup.

As discussed above, the fact that only the space bisection task results improved after room observation suggests that the transfer of information from the visual system toward the auditory one occurs only for those aspects for which the visual system can be used to calibrate the auditory one. In this vein, gaining knowledge about room acoustics through vision seems to be involved much more when estimating complex relationship between sound sources: while estimating audible angles requires comparison between the estimated direction of two sound sources, space bisection requires establishing a specific ordering relation between the direction of three sound sources, of which two are far apart in space. This operation may require Euclidian representation of space (Gori et al., 2014) and involve more spatial processing, possibly related to cues linked to the room structure that visual input helps to interpret.

A final interesting result is that no difference was observed between the performance obtained for the space bisection task after 1 min of environment observation and in the condition in which the eyes were maintained open for the entire experimental session. This suggests that the visual system needs only a brief period of environment observation to allow an improvement in this audio task.

In our past works we suggested that a process of cross-modal calibration might occur during development (Gori et al., 2008). During this process the visual system seems to be involved in the calibration of auditory space bisection (Gori et al., 2012, 2014). A possible interpretation of the results presented in this paper is that vision can calibrate audition also in a short-term form in adult individuals. It can indeed improve auditory space precision through a transfer of information about environmental cues from the visual system. In particular our results suggest that visual information might help the hearing system to compensate for the mismatch produced by echoes, and that visual knowledge of the room structure is linked to understanding of room acoustics.

If this interpretation is correct, then our results can be discussed in relation to the echolocation technique. Some blind individuals use the echoes produced by the environment to their advantage, thanks to echolocation. Human echolocation is an ability of humans to detect objects in their surroundings by sensing echoes from those objects. By actively creating sounds, such as clicks produced by rapidly moving the tongue in the palatal area behind the teeth (Rojas et al., 2009) or sounds produced by external mechanical means such as tapping a cane against the floor (Burton, 2000), people trained to orientate with echolocation can interpret the sound waves reflected by nearby objects. Many studies conducted under controlled experimental conditions have shown that echolocation improves blind people’s spatial sensing ability.

For example a recent study (Vercillo et al., 2015) has compared the performance of expert echolocators, blind and sighted people with no previous experience of echolocation, in a space bisection task. It was found that blind expert echolocators performed the spatial bisection task with similar or even better precision than the sighted group. Moreover, in several studies were demonstrated that echolocation improves the ability to determine other characteristics as distance (Kolarik et al., 2013), motion (Thaler et al., 2011, 2014), size (Teng and Whitney, 2011; Teng et al., 2012), shape (Thaler et al., 2011; Milne et al., 2014).

Therefore we can assume that echolocation could serve to recalibrate the ability of blind individuals to represent sounds in some spatial configurations and compensate the lack of vision. Our results support the idea that the visual system might in some form compensate for the mismatch produced by echoes in unknown environments by helping to interpret them. Visual information and spatio-acoustic representation appear therefore intertwined. If this is correct then the use of the echolocation technique can be a way of substituting the role of the visual system on this process. This would partially explain the improved spatial skills of blind expert echolocators. To conclude, the current findings suggest that vision is not only important for the auditory system during the development of space auditory representation, but also during adulthood. Although the mechanisms that subtend this process still have to be completely understood, our results suggest that the visual system can improve some forms of auditory spatial perception also in adults and after short-term environmental observation.

## Conflict of Interest Statement

The authors declare that the research was conducted in the absence of any commercial or financial relationships that could be construed as a potential conflict of interest.
